# The Efficacy of Immune Checkpoint Inhibitors in Microsatellite Stable Colorectal Cancer: A Systematic Review

**DOI:** 10.1093/oncolo/oyae013

**Published:** 2024-02-03

**Authors:** Deniz Can Guven, Gozde Kavgaci, Enes Erul, Masood Pasha Syed, Tara Magge, Anwaar Saeed, Suayib Yalcin, Ibrahim Halil Sahin

**Affiliations:** Department of Medical Oncology, Hacettepe University Cancer Institute, Ankara, Turkey; Health Sciences University, Elazig City Hospital, Elazig, Turkey; Department of Medical Oncology, Hacettepe University Cancer Institute, Ankara, Turkey; Department of Internal Medicine, Hacettepe University Faculty of Medicine, Ankara, Turkey; Division of Hematology/Oncology, Department of Medicine University of Pittsburgh School of Medicine Pittsburgh, Pittsburgh, PA, USA; Division of Hematology/Oncology, Department of Medicine University of Pittsburgh School of Medicine Pittsburgh, Pittsburgh, PA, USA; Division of Hematology/Oncology, Department of Medicine University of Pittsburgh School of Medicine Pittsburgh, Pittsburgh, PA, USA; Department of Medical Oncology, Hacettepe University Cancer Institute, Ankara, Turkey; Division of Hematology/Oncology, Department of Medicine University of Pittsburgh School of Medicine Pittsburgh, Pittsburgh, PA, USA

**Keywords:** colorectal cancer, immunotherapy, immune checkpoint inhibitors, microsatellite stable, tyrosine kinase inhibitors

## Abstract

The use of immune checkpoint inhibitors (ICIs) has revolutionized cancer care, particularly in immune-inflamed tumors and tumors with a high mutational burden, like microsatellite instable colorectal cancer (CRC). However, their effectiveness in microsatellite stable (MSS) CRC is limited. This systematic review aims to evaluate the efficacy of ICIs in MSS CRC and explore promising combination strategies. A comprehensive search from the Web of Science, Medline, and Embase databases, for studies published until 14 November 2022, identified 53 clinical trials included in the review. ICI monotherapy or ICI-ICI combinations demonstrated limited clinical activity for patients with MSS CRC, with overall response rates below (ORR) 10% in most studies. The ICI and tyrosine kinase inhibitor (TKI) garnered ORRs ranging from 10% to 40% and indicated a higher benefit for patients, particularly those without active liver metastases. The combination of ICIs with anti-VEGF agents showed modest ORRs, especially in the earlier treatment lines and in combination with chemotherapy. While these combinations could lead to modest improvements, well-defined biomarkers for long-term benefit are yet to be delineated. Combinations involving BRAF inhibitors with ICIs were studied, showing promising responses with combination approaches in molecularly defined subgroups. In conclusion, while ICI monotherapy has limited efficacy in MSS CRC, combination strategies hold promise to enhance survival outcomes. Further research is necessary to identify optimal combination approaches, predictive biomarkers for treatment response, as well as enrollment according to tumor molecular characteristics.

## Teaser

This review evaluates the efficacy of immune checkpoint inhibitors in ICIs in microsatellite stable colorectal cancer and explores promising combination strategies.

**Implications for Practice**

This systematic review underscores the evolving landscape of immunotherapy in colorectal cancer (CRC), particularly highlighting its limited efficacy in microsatellite stable (MSS) CRC. While immune checkpoint inhibitors (ICIs) alone have shown modest benefit, promising responses emerge when combined with targeted therapies like tyrosine kinase inhibitors (TKIs) and BRAF inhibitors. Additionally, combinations with anti-VEGF agents and chemotherapy warrant consideration, especially in earlier treatment lines. However, the need for well-defined biomarkers to guide treatment selection and predict long-term benefits remains paramount. This review calls for continued research to refine combination strategies and enhance patient outcomes in MSS CRC based on tumor molecular characteristics.

## Introduction

Immunotherapy became the fifth pillar of cancer care and significantly changed the landscape of cancer management in the last decade.^1^ The immune checkpoint inhibitors (ICIs), a class of immunotherapy agents, work by releasing the breaks of immune systems with the modification of regulatory immune checkpoints and paved the way in the immunotherapy era.^2,3^ The benefit of ICIs is particularly prominent for patients with immune-inflamed tumors like melanoma, non-small cell lung cancer, and renal cell carcinoma, as well as tumors with high mutation burden with increased neo-antigen loads.^4-8^ In contrast, the ICIs had limited efficacy for patients with immune-cold tumors and tumors with a lower mutational burden.^9,10^

Microsatellite instability-high (MSI-H) colorectal cancer (CRC) was among the earliest tumors in which a benefit with ICIs was demonstrated. In the seminal paper by Le et al., the response rate to pembrolizumab monotherapy was 40% in mostly pretreated patients with CRC,11 and in the update of this study by expansion cohorts, the response rate was over 50% in patients with metastatic MSI-high CRC. This benefit was confirmed in larger-scale studies and in long-term follow-ups, suggesting highly durable disease control for patients with MSI-H CRC with ICIs.^12,13^ The MSI-H CRC carry a higher tumor mutation burden (TMB) with a median TMB of 46.8 mutations/megabase, and this high TMB due to frameshift alterations leads to increased neoantigen generation and expression representing the hallmark of MSI-H CRC for high ICI efficacy.^14^

While the ICIs became the standard of care for patients with MSI-H CRC, earlier studies demonstrated very limited ICI benefits for patients with MSS CRC, especially with ICI monotherapy.^11,15^ The MSS CRC has distinct molecular pathogenesis and tumor microenvironment (TME) compared to MSI CRC.^16,17^ The tumor mutational burden is significantly lower in MSS CRC than in MSI-H CRC.^14^ Additionally, immunosuppression and immune exhaustion in TME are more prominent in MSS CRC.^18^ Despite these inherent limitations for the clinical benefit of ICIs for patients with MSS CRC, there is an important body of data in MSS CRC for the use of ICIs, albeit with variable designs, sample sizes, and treatment lines. Several combination strategies with tyrosine kinase inhibitors (TKIs), vascular endothelial growth factor inhibitors (anti-VEGF), and chemotherapy have been tested in clinical trials and yielded varying results. Therefore, in this article, we systemically reviewed recently published clinical trials on the ICI efficacy in MSS CRC to overview the promising combination strategies and review the ongoing studies to give perspectives on how the field could evolve.

## Methods

### Search Strategy

We report this systematic review following the Preferred Reporting Items for Systematic Reviews and Meta-analyses (PRISMA) 2020 guideline.^19^ This protocol was registered with the Open Science Framework at https://doi.org/10.17605/OSF.IO/GZ7RX (accessed on July 11, 2023). We systematically searched the Web of Science (topic), Medline (all fields), and Embase (all fields) databases for studies published until 14 November 2022 for inclusion. The selected MeSH search terms were (“colon cancer” OR “colorectal cancer” OR “rectum cancer” OR “rectal cancer”) AND (“immunotherapy” OR “immune checkpoint inhibitor”) AND (“microsatellite stable”). Additionally, congress abstracts from the ESMO and ASCO congresses were evaluated for inclusion.

### Study Selection and Data Extraction

We included clinical trials evaluating the ICI efficacy in advanced MSS CRC. In order to prevent missing any relevant studies, studies without the available full-text publication were included. The exclusion criteria were as follows: duplicated articles, animal studies, commentaries, opinion papers, and reviews, articles not in English, studies without separate data for MSS CRC patients, the inclusion of the patients with localized disease, studies from real-life cohorts, and study protocols of ongoing trials.

DCG and EE independently screened the literature to assess their eligibility for inclusion in the study. Two authors (DCG and EE) independently extracted the following data from the studies following the PRISMA 2020 guidelines: clinical trial number, treatment line, treatment scheme, overall response rate (ORR), disease control rate (DCR), median progression-free survival (PFS), and median overall survival (OS). The ORR was defined as the proportion of patients who had either confirmed complete or partial response, and the DCR was defined as the proportion of patients who had a complete or partial response or stable disease under ICIs. Any disagreements were resolved by discussing with the senior author (IHS). The individual study qualities were evaluated with the MINORS tool for single-arm studies^20^ and Risk of Bias (RoB) version 2 for randomized trials.^21^

## Results

### Article Selection

**Figure 1.**
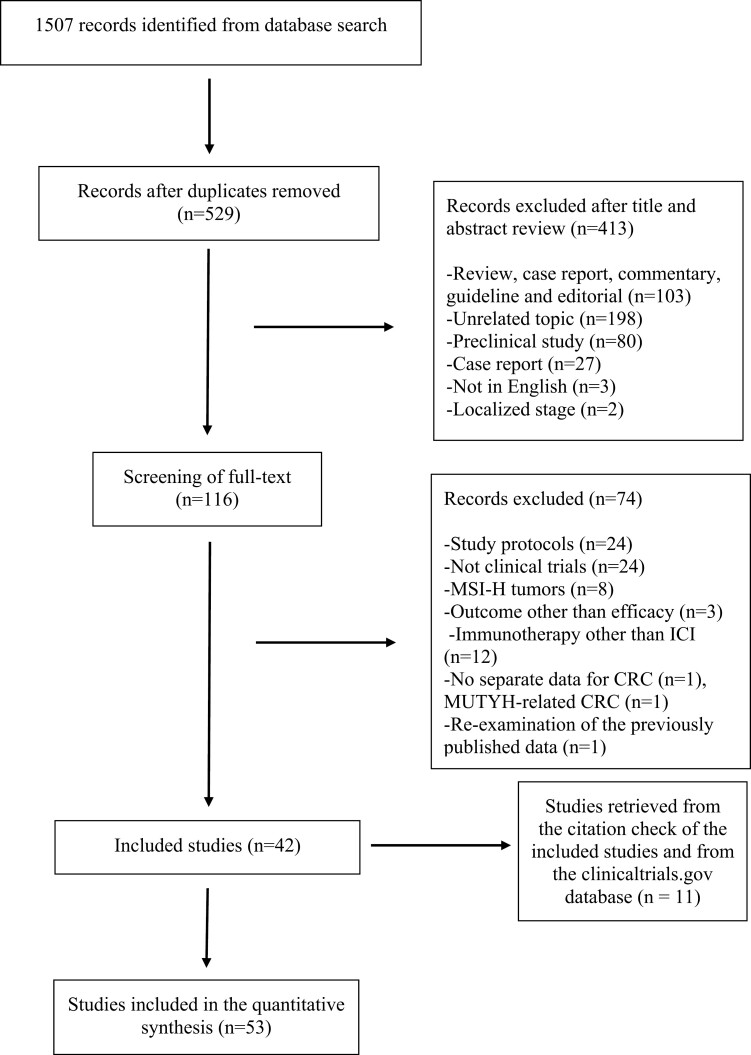
PRISMA flow diagram.

A total of 1507 records were identified with a database search. After removing the duplicates (*n* = 978), we then excluded 413 records after screening the titles and abstracts. The full texts of the remaining 116 records were evaluated, and additional 74 records were excluded due to the following reasons: study protocols (*n* = 24), not clinical trials (*n* = 24), MSI-H tumors (*n* = 8), outcome other than efficacy (*n* = 3), immunotherapy other than ICI (*n* = 12), no separate data for CRC (*n* = 1), MUTYH-related CRC (*n* = 1), and re-evaluation of the previously published data (*n* = 1). Eleven additional studies were retrieved from the citation check of the included studies and from the clinicaltrials.gov database. Fifty-three clinical trials were included in the quantitative synthesis. The PRISMA diagram for article selection is summarized in Fig. 1.

### Immune Checkpoint Inhibitor Monotherapy or Doublets

A total of 5 studies evaluated the use of ICI therapy alone, either as monotherapy or ICI-ICI combinations in MSS CRC (Table 1). One study was phase I^23^, and the remaining 4 studies were phase II.^11,12,22,24^ No phase III studies were available. Two studies enrolled patients who had received the 3+- prior lines of therapy. The sample sizes varied between 18 and 180. The ORR was the main outcome in most studies. The ORR was consistently < 10% in all studies except the botensilimab plus balstilimab combination.^23,25^ The DCR varied between 11% and 73%. ICI monotherapy has not shown any significant clinical activity, and no objective responses were seen in 2 studies with pembrolizumab or nivolumab.^11,22^ However, the proportion of patients on nivolumab for 18 weeks and beyond was 38% in patients without liver metastases in the ANICCA study testing nivolumab monotherapy in tumor-specific major histocompatibility complex II (tsMHC-II)-positive MSS CRC.^22^

**Table 1.** Immune checkpoint inhibitor monotherapy or dual immune checkpoint inhibitors.

Clinical trial identifier/study title	Treatment line	Treatment scheme	Sample size (*n*)	Phase/(randomization)	ORR	DCR	mPFS (months)	mOS (months)	Additional comments
NCT03981146/ ANICCA^22^	N/A (No SOC option and had tumors with >1% tsMHC-II)	Nivolumab	35	2	0%	8.6%	9.1 weeks	29.6 weeks	The proportion of patients with liver metastases on nivolumab for 18 weeks and beyond was 9%, while this rate was 38% in patients without liver metastases. The rate for tsMHC-II-positive tumors was 13% in screened patients with MSS CRC
NCT03860272^23^	≥1 prior line of treatment	Botensilimab + balstilimab	59	1	22%	73%	4.1	NR (12-month OS rate 61%)	The benefit persisted in the updated data. The updated data from the World GI Congress (*n* = 87): ORR in patients without liver mets 23%, ORR in patients with liver mets 0%
NCT02870920/ The Canadian Cancer Trials Group CO.26 Study^24^	All patients had received multiple lines of prior chemotherapy	Tremelimumab + durvalumab	119	2 (2:1)	1.2%	22.7%	1.8	6.6	Grades 3-4 AEs in 64% of the patients treated with ICIs Patients with plasma TMB of 28/Mb or higher (21% of the patients with MSS CRC) had OS benefit (HR: 0.34, 90% CI: 0.18-0.63)
		Best supportive care alone (BSC)	61		0%	6.6%	1.9	4.1	
NCT01876511/ KEYNOTE-016^11^	≥ 2 previous lines of chemotherapy	Pembrolizumab	18	2	0%	11%	2.2	5	
NCT02060188/ CheckMate 142^12^	≥1 prior line of treatment	Nivolumab	23	2	N/A	N/A	1.4	N/A	

Clinical trials assessing dual ICIs have been tested. The ORR and DCR of phase I study of botensilimab (next generation CTLA-4 inhibitor) plus balstilimab (PD-1 inhibitor) combination were 22% and 73%, respectively.^23^ Importantly, in the updated data from this study, the ORR in patients with or without active liver metastases was 0% and 23%, respectively. In a randomized phase II trial of durvalumab plus tremelimumab combination with best supportive care as the control arm, there was one patient with MSS tumor who showed a partial response (PR) lasting more than 21 months.^24^ In the same study, patients with high TMB (28 Mb or higher) derived the greatest OS benefit from ICI therapy (HR, 0.34; 90% CI, 0.18-0.63; *P* = .004).

### Immune Checkpoint Inhibitor and Tyrosine Kinase Inhibitor Combination

Ten studies tested the efficacy of the ICI plus TKI combination in MSS CRC (Table 2). Regorafenib was the most commonly used agent in these studies. The recommended phase II dose for regorafenib was 80-120 mg in the available studies. The available studies yielded mixed results. The Regonivo study conducted in Japan included gastric and patients with CRC.^26^ For the patients with MSS CRC, the ORR was 33.3%, while it was 7% in a similarly designed study from the US.^31^ Additionally, 63.6% of the lung target lesions responded, while the response rate in target liver lesions was 8.3% in the Regonivo study. Similarly designed Regomune study with regorafenib and avelumab reported no response in 48 patients.^33^ The median PFS varied between 2.3 months in the LEAP-005 study with pembrolizumab and lenvatinib to 7.9 months in the Regonivo study.^26,34^ Grade 3 or higher adverse events were variable, with studies reporting up to 90% grade 3 or higher adverse events with combination were present.

**Table 2.** Immune checkpoint inhibitor and tyrosine kinase inhibitor combination.

Clinical trial identifier/study title	Treatment line	Treatment scheme	Sample size (*n*)	Phase (randomization)	ORR	DCR	mPFS (months)	mOS (months)	Additional comments
NCT03406871 (REGONIVO/EPOC1603)^26^	≥2 previous lines of chemotherapy	Regorafenib + nivolumab	25	1/2	33.3%	N/A	N/A	NR	63.6% of the lung target lesions responded, while the response rate in target liver lesions was 8.3%
NCT03712943^27^	≥2 previous lines of chemotherapy	Regorafenib + Nivolumab	50	1	10% (40 evaluable patients)	63% (40 evaluable patients)	4.3	11.1	PR rates in patients with and without liver metastases were 4% and 25%, respectively.
ChiCTR2000028965^28^	Third line	Fruquintinib + toripalimab	30	2	16.7%	54.1%	6.0	8.0	A single-center study. 25% Grade 3-4 TRAE was reported.
NCT03912857	Third line	SHR-1210 (an anti-PD-1 antibody) + apatinib		2	0%	22.2%	1.83	7.80	All patients experienced treatment-related adverse events (AEs). The unacceptable toxicity of SHR-1210 coupled with apatinib may be the primary reason why the efficacy of treating MSS mCRC has not improved.
NCT04695470^29^	Patients who failed at least second-line standard chemotherapies	Fruquintinib + sintilimab	10	2	16%	77%	4.1	13.3	High rates of hypertension (70%) and proteinuria (70%). Grade 3 AEs in 90% of the patients
NCT03539822/ CAMILLA^30^	≥2 prior line of treatment (CRC)	Cabozantinib + durvalumab	17	1/2	23.5%	88.2%	4.6	9.6	Basket study including patients with CRC, GEA, and HCC. The ORR was 41.67% in CPS ≥ 5 patients. Higher PFS in patients with CPS ≥ 5 high tumors and less tumor-associated macrophages and greater tumor-infiltrating CD4 T cells
NCT04126733^31^	≥2 previous lines of chemotherapy	Regorafenib + nivolumab	70	2	7%	39%	1.8	11.9	All responders had no liver metastases at baseline. Grade 5 AEs in 3% of the patients
NCT03657641^32^	≥1 prior line of treatment	Regorafenib + Pembrolizumab	73	1/2	0%	49%	2	10.9	Largest trial of ICI + regorafenib in MSS CRC The median PFS in patients without liver metastases was 4.3 months
NCT03475953/REGOMUNE^33^	≥1 prior line of treatment	Regorafenib + avelumab	48	2	0% (43 evaluable patients)	54% (43 evaluable patients)	3.6	10.8	Shorter PFS and OS in patients with higher baseline tumor-associated macrophages Better survival in patients with increased CD8 + T-cell infiltration in cycle 2
NCT03797326/ LEAP-005^34^	≥ 2 previous lines of chemotherapy	Lenvatinib + Pembrolizumab	32	2	22%	47%	2.3	7.5	Despite these promising results, the press release from the phase III LEAP-017 study demonstrated no PFS or OS benefit with the combination

### Immune Checkpoint Inhibitor and Chemotherapy Combinations

Eight studies evaluated the efficacy of chemotherapy and ICI combinations (Table 3). Three studies were conducted in the 2nd line or later with capecitabine or trifluridine-tipiracil (TPI), while 4 studies were conducted in the first line with chemotherapy combinations. Five of the studies used bevacizumab in combination with chemotherapy and ICIs. While the frontline AtezoTRIBE and Checkmate 9 × 8 studies were randomized phase II studies, the remaining studies were single-arm phase I/II studies.^36,44^ These randomized studies conducted in the 1st-line included patients with MSI-H and MSS CRC and used PFS as the primary endpoint. Both studies were negative regarding the primary outcome in the MSS subgroup. In the Checkmate 9 × 8 study, subgroup analyses according to CMS subtypes demonstrated that in patients with CMS1 or CMS3 disease, there was a PFS benefit after 12 months of follow-up.44 Lastly, the preliminary results of the MEDITREME trial, in which the combination of FOLFOX with durvalumab and tremelimumab is being investigated as first-line therapy, showed an ORR of 61%, and 8.4 months PFS43 among patients with KRAS-mutated CRC.

**Table 3.** Immune checkpoint inhibitor and chemotherapy combinations.

Clinical trial identifier/study title	Treatment line	Treatment scheme	Sample size (*n*)	Phase/(randomization)	ORR	DCR	mPFS (months)	mOS (months)	Additional comments
NCT02848443^35^	≥1 prior line of treatment	Trifluridine/tipiracil + oxaliplatin ± nivolumab or bevacizumab	17	1	7.1 (14 evaluable patient)	71.4	6	NR	The study enrollment was stopped due to less-than-expected ORR
NCT03721653/ AtezoTRIBE^36,37^	First line	FOLFOXIRI + bevacizumab	73	2 (1:2)	64	93	11.5	N/A	Included both MSI-H and MSS patients. PFS subgroup analyses for MSS patients 11.4 vs. 12.9 months, *P* = .071
		FOLFOXIRI + bevacizumab + atezolizumab	145		59	92	13.1	N/A	
NCT05171660^38^	First-line	Sintilimab (IBI 308) + CAPEOX + bevacizumab (BBCAPX)	25	2	84%	100%	NR	N/A	All patients with lung metastases had a response (*n* = 4). The ORR in patients with liver metastases was 93.3%
NCT02873195/ BACCI^39^	Patients progressed on 5-FU, oxaliplatin, irinotecan, bevacizumab, and anti-EGFR therapy (if RAS wt). Prior anti-PD-1/PD-L1 therapy was not permitted.	Capecitabine, bevacizumab + placebo	46	2 (1:2)	4.4%	N/A	3.3	N/A (12-month OS 43%)	89.4% of the study population had MSS CRC The PFS HR in the MSS subgroup was 0.66 (95% CI: 0.44-0.99) Higher response rate and OS in patients without liver metastases in the atezolizumab arm (ORR 23.1 vs. 5.8%, HR for OS 0.33 vs. 1.14)
		Capecitabine, bevacizumab + atezolizumab	82		8.54%	N/A	4.4	N/A (12-month OS 52%)	
NCT03832621/ MAYA^40^	Patients with pretreated mCRC (MGMT Silenced)	Temozolomide Followed by Ipilimumab + Nivolumab	135	2	45%	N/A	7.0	18.4	29% of the molecularly screened patients had MGMT silencing -Implied a novel design with ICI maintenance after non-progression with 2 cycles of temozolomide induction.
NCT02860546^41^	Patients with histologically proven MSS mCRC and disease progression after ≥ 2 prior chemotherapy	Trifluridine/tipiracil (FTD/TPI) + nivolumab	18	2	0%	56%	2.8	N/A	No dose-limiting toxicities. Did not progress to efficacy stage after safety lead-in
NCT02811497^42^	Patients must have progressed or be intolerant of 5-FU, irinotecan, oxaliplatin and epidermal growth factor receptor (EGFR) mAb in patients with RAS wild-type tumors, in recurrent/metastatic setting.	Azacitidine + Durvalumab	28 (15 MSS CRC)	2	0 (all cohort)	7.1% (all cohort)	1.9 (all cohort)	5 (all cohort)	Basket study including patients with MSS CRC, breast cancer and platinum-resistant ovarian cancer -No response in 15 patients with MSS CRC
NCT03202758/ MEDITREME^43^	First line (RAS mutated)	mFOLFOX6 (6 cycles) + durvalumab + tremelimumab	57	1/2	61%	89%	8.4	N/A	Included 3 patients with MSI-H disease
NCT03414983/ Checkmate 9 × 8^44^	First line	Nivolumab + mFOLFOX6 + bevacizumab (SOC)	127	2 (2:1)	60%	91%	11.9	29.2	Included both patients with MSS and MSI-H CRC No PFS benefit with the combination, although higher PFS rates after 12 months
		SOC	68		46%	84%	11.9	NR	

### Other Combinations

In addition to ICI monotherapy, ICI-TKI, and ICI-chemotherapy combinations, several other agents were evaluated in combinations (Table 4). The BRAF or MEK inhibitors were tested in combination with ICIs in 4 studies in patients with MSS CRC. In one of the earliest studies, the combination of encorafenib, cetuximab, and nivolumab was related to an ORR of 45% in patients with chemotherapy-refractory unresectable or metastatic MSS, BRAF-V600E mutated CRC, while the combination of atezolizumab and cobimetinib was related to an ORR of 3% in patients with previously treated metastatic MSS CRC in the IMblaze 370 study.^51,67^ Five additional studies evaluated the efficacy of radiotherapy plus ICI combinations. The NCT03104439 study evaluating radiotherapy plus nivolumab and ipilimumab in patients with metastatic MSS CRC demonstrated 15% ORR and 2.5 months PFS. The study included heavily pretreated patients, with a median of 2 previous treatment lines.^62^ In the pooled analysis of the MSS CRC cohort of 2 PraG basket studies, the combination of radiotherapy and anti-PD-1 inhibitor and GM-CSF, the ORR was 25%, and the DCR 62.5%.^66^ Additionally, one of 9 treated patients had complete response. Several other agents, such as EGFR antagonists, immunomodulatory agents, BITE, and CART therapies, were also tested with ICIs, and the results of these studies are summarized in Table 4.

**Table 4.** Other Combinations

Clinical trial identifier/study title	Treatment line	Treatment scheme	Sample size (n)	Phase (randomization)	ORR	DCR	mPFS (months)	mOS (months)	Additional comments
NCT01988896^45^	N/A	Atezolizumab + cobimetinib	84	1	8% (6/62 in MSS CRC patients)	N/A	The 12-month PFS rate was 11%	9.8	Included 2 patients with MSI-H disease and 20 patients with unknown MS status
NCT03152565 (GEMCAD1602)^46^	≥ 2 prior lines of treatment	Avelumab + vaccines with autologous dendritic cells (ADC)	19	1/2	0%	22%	3.1	12.2	Hiperprogressive disease in 4/18 of patients with response evaluation
EPOC1704^47^	≥1 prior line of treatment	TAS-116 (Pimitespib) + Nivolumab	29 (1 MSI-H patient)	1	14%	84%	3.0	10.1	The ORR was 16% in patients with MSS CRC without prior ICI exposure. ORR was 27% in patients with CPS ≥ 1, and 0% in patients with CPS < 1 ORR was 33% with TMB-high (median as the cutoff) and 12% with TMB low
EPOC1503/SCOOP^48^	≥1 prior line of treatment	Napabucasin + pembrolizumab	40	1/2	10.0%	45.0%	1.6	7.3	The ORR was 0%, 5.3%, and 42.9% in CPS < 1, 1 ≤ CPS < 10, and CPS ≥ 10, respectively
NCT02636036 (SPICE)^49^	≥1 prior line of treatment	Enadenotucirev (EnAd) + nivolumab	45 (All cohort 51 with 6 HNC cases)	1	2.4 (2.1 in all cohort)	46.3 (46.8 in all cohort)	1.6	16	An increase in CD8 + T-cell infiltration with treatment in 12 of 14 patients with matching biopsies
NCT03206073^50^	≥ 2 previous lines of chemotherapy	Pexastimogene devacirepvec (PexaVec) + durvalumab	16	1/2	6.3%	12.5%	2.3	7.5	Treatment discontinuation due to toxicities in 4/22 patients in the PexaVec/durvalumab/tremelimumab arm An increase in the ICOS + Ki-67 + CD8 + and PD1 + Ki-67 + CD8 + T-cells with treatment
		Pexastimogene devacirepvec (PexaVec) + durvalumab + tremelimumab	18		0%	16.7%	2.1	5.2	
NCT04017650^51^	≥1 prior line of treatment	Encorafenib + cetuximab + nivolumab	26	1/2	45% (21 patients evaluable for response)	95% (21 patients evaluable for response)	7.3	11.4	A randomized prospective trial (SWOG 2107) is planned due to these encouraging results
NCT02298959^52^	≥1 prior line of treatment	Ziv-aflibercept + pembrolizumab	6 (All cohort 33)	1	0%	0%	2.5	3.3	
NCT02650713	No available SOC option	RO6958688 (novel T-cell bispecific antibody targeting CEA) + atezolizumab	14	1	21.5%	50%	N/A	N/A	Metabolic partial response in PET imaging in 7/14 patients with combination Enhanced activity with CEA-TCB and ICI combination than the CEA-TCB monotherapy
NCT03168139 KEYNOTE-559/OPERA^53^	No available SOC option	NOX-A12 (Olaptesed) + Pembrolizumab	11	1/2	0	27	N/A	N/A	Th1-like reactivity with CXCL12 inhibition (NOX-A12)
NCT02503774^54^	≥1 prior line of treatment	Oleclumab + durvalumab	54	1	2.4%	23.8% (DCR at least for 8 weeks)/4.8% (DCR for at least 24 weeks)	6-month PFS rate 5.4%	7	In all 3 patients with MSS CRC or PDAC and had a response to treatment, ≥ 80% of tumor cells expressed CD73
NCT02837263^55^	≥1 prior line of treatment	Stereotactic body radiotherapy (SBRT) + Pembrolizumab	15	1	N/A	1-year RFS was 67% (historic control 50%)	N/A	N/A	All patients had resectable liver-confined metastatic MSS CRC Study Design: Sequential SBRT and 1 cycle pembrolizumab-Surgery-Adjuvant Pembrolizumab Two patients with BRAF V600E mutation and 2 patients with VCAN high tumors had early recurrences
ACTRN12617001573347^56^	≥1 prior line of treatment	Pixatimod + nivolumab	7 (All cohort 23)	1	0%	28%	N/A	N/A	
NCT03428126^57^	≥1 prior line of treatment	Durvalumab + trametinib	29	2	3.4%	27.4%	3.2	6.9	Discrepant response according to metastasis site in 3 patients (responses in the lung mets, and no response in liver lesions) No changes in T-cell infiltration but upregulation of PD-1 and Tim3 on CD8 T-cells in on-treatment biopsies
NCT04332653^58^	No SOC treatment option	NT-I7 (efineptakin alfa) + pembrolizumab	28	1/2	12%	N/A	N/A	N/A	An increase in peripheral blood lymphocytes with treatment
NCT03007407/ NSABP FC-9^59^	1-2 prior lines of therapy	Durvalumab (D) + tremelimumab (T) following palliative hypofractionated radiotherapy (SBRT)	33	2	10% (20 evaluable patients)	20% (20 evaluable patients)	N/A	N/A	Study design: SBRT (3*9 Gy)-T + D for 4 cycles-Durvalumab monotherapy for 5-12^th^ cycles
NCT03442569/ LCCC1632^60^	1-2 prior lines of therapy	Ipilimumab, nivolumab + panitumumab	56	2	35 (49 evaluable patients)	N/A	5.7	N/A	Included patients with KRAS/NRAS/BRAF WT disease. No use of anti-EGFR inhibitors previously
NCT03475004^61^	At least 2 prior lines of therapy	Pembrolizumab, binimetinib + bevacizumab	50	2	13% (39 evaluable patients)	74%	5.8	N/A	
NCT03104439^62^	No SOC treatment option	RT, ipilimumab + nivolumab	40	2	15%	37%	2.5	10.9	DCR was 25% in all cohort, and 37% in the per-protocol population (received RT in addition to ICIs). Higher expression of NK cells and the HERVK repeat RNA in patients with disease control
NCT03050814^63^	First-line	FOLFOX/bevacizumab	10	2 (1:1)	50%	N/A	8.8	18.6	The 8/11 patients in the experimental arm had multifunctional CD4+/CD8 + T-cell responses to cascade antigens MUC1 and/or brachyury. This did not lead to a survival benefit
		FOLFOX/bevacizumab ± CEA-targeted vaccine/avelumab	16		56.3%	N/A	10.1	15.1	
NCT03993626/ CAROSELL^64^	At least 2 lines of systemic anticancer therapies	CXD101 + nivolumab	55	1/2	9% (46 evaluable patients)	48% (46 evaluable patients)	2.1	7.0	Met its’ primary endpoint (DCR) -14.5% of the patients were alive at 2 years
NCT03005002^65^	MSS metastatic CRC with liver‐predominant disease who progressed following at least one prior line of treatment	Yttrium‐90 Liver radioembolization followed by durvalumab + tremelimumab	9	1	0	0	N/A	N/A	Tumor biopsies showed low levels of TIL before and after Y90 radioembolization
NCT04892498 PRaG^66^	No SOC treatment option	PD-1 Inhibitor Combined with Radiotherapy and GM-CSF (PRaG)	12	2	25%	62.5%	4.7	N/A	Used a combination of RT-anti-PD1-G-CSF/GM-CSF and IL-2
NCT02788279/ IMblaze 370^67^	Third-line	Atezolizumab + cobimetinib	183	3 (2:1:1)	3%	26%	N/A	8·87	Included 6 patients with MSI-H disease
		Atezolizumab	90		2%	19%	N/A	7·10	
		Regorafenib	90		2%	31%	N/A	8·51	
NCT02888743/ ETCTN 10021^68^	≥1 prior line of treatment	Durvalumab + tremelimumab + RT	20	2	0%	5.5%	N/A	N/A	Stable disease in out-of-field lesions in one patient (18 evaluable patients) -Correlative IF revealed that HFRT, but not LDFRT, increased infiltration of CD8+ and CD8+/PD1+/Ki67 + T cells in the RT field
NCT02349724^69^	≥1 prior line of treatment	Anti-CEA-CAR T	10	1	0%	70%	N/A	N/A	No CAR-T related severe adverse events -Long-term disease control in 2 patients -Persistence of CAR-T T cells in peripheral blood and tumor tissues
NCT04561336 CAVE CRC^70^	First-line	Avelumab + cetuximab	77	2	7.8%	65%	3.6	11.6	
NCT03724851^71^	≥1 prior line of treatment	Vactosertib + pembrolizumab	33	2	15.2%	36.3%	N/A	N/A	- 82% were consensus molecular subtype (CMS) 4.
NCT03274804/ PICCASSO^72^	≥1 prior line of treatment	Maraviroc + pembrolizumab	20	1	5.3%	5.3%	2.1	9.83	Despite pembrolizumab in combination with maraviroc demonstrated feasibility and exhibited a favorable toxicity profile, clinical activity was limited

### Ongoing Studies

Our search retrieved a total of 38 clinical trial records (Table 5). Five studies did not start recruiting participants at the time of this review. Twenty of the studies were active and recruiting. Thirteen of the studies were ongoing, and participants were receiving an intervention or being examined, but potential participants were not currently being recruited or enrolled. There were 4, 31, and 3 phases I, II, and III studies, respectively. Seven of these trials were first-line studies. The ICI and TKI combinations and chemotherapy and ICI combinations are being evaluated in 6 and 10 studies, respectively. Additionally, 17 studies evaluated other TME modifiers in combination with ICIs, while RT and ICI combinations were evaluated in 5 studies. The sample sizes varied between 12 and 665, and 7 studies were of the Far East region.

**Table 5.** Ongoing clinical trials from the clinicaltrials.gov database.

Study title	Clinical trial identifier	Phase	Treatment arm(s)	Recruitment status	Estimated (actual) enrolment	Primary outcome
Nivolumab and ipilimumab and radiation therapy in metastatic, microsatellite stable colorectal cancer	NCT04575922	2	Nivolumab + Ipilimumab + RT	Active, not recruiting	30 (32)	ORR for unirradiated lesions
A phase II trial assessing nivolumab in class II expressing microsatellite stable colorectal cancer (ANICCA)	NCT03981146	2	Nivolumab	Active, not recruiting	36 (35)	Durable clinical benefit
Nivolumab and ipilimumab and radiation therapy in MSS and MSI high colorectal and pancreatic cancer^62^	NCT03104439	2	Nivolumab + ipilimumab + RT	Recruiting	80	DCR
Study of TBio-6517 Given Alone or in Combination With Pembrolizumab in Solid Tumors (RAPTOR)	NCT04301011	1/2	TBio-6517 TBio-6517 +Pembrolizumab	Active, not recruiting	84 (27)	Incidence of AEs MTD and determination of the recommended phase II dose ORR
Chemotherapy and immunotherapy as treatment for MSS metastatic colorectal cancer with high immune infiltrate (POCHI)	NCT04262687	2	XELOX + bevacizumab + pembrolizumab	Recruiting	55	PFS at 10-months
A phase Ib/IIa study of CR6086 in combination with balstilimab in pMMR-MSS metastatic colorectal cancer patients	NCT05205330	1/2	CR6086 (30/90/180 mg) + AGEN2034	Active, not recruiting	27 (28)	Safety and tolerability DCR
Study of safety and efficacy of DKY709 alone or in combination with PDR001 in patients with advanced solid tumors.	NCT03891953	1	DKY709 DKY709 + PDR001 (Spartalizumab)	Active, not recruiting	320 (99)	Safety and tolerability Incidence of DLTs
A study of nivolumab alone or nivolumab combination therapy in colon cancer that has come back or has spread (CheckMate142)^12^	NCT02060188	2	Nivolumab nivolumab + ipilimumab nivolumab + ipilimumab + cobimetinib nivolumab + BMS-986016 nivolumab + daratumumab	Active, not recruiting	96 (385)	ORR
Pembrolizumab and autologous dendritic cells for the treatment of refractory colorectal cancer (CRC)	NCT05518032	2	Pembrolizumab + autologous dendritic cells	Not yet recruiting	20	ORR
A dose escalation/expansion study of MDK-703 in patients with advanced or metastatic solid tumors (ORCHID-1)	NCT05716295	1/2	MDK-703 MDK-703 + immune checkpoint inhibitor	Recruiting	150	DLTs/MTD Optimal biological dose Recommended dose Incidence and severity of TEAEs and serious AEs
Study of nivolumab and relatlimab in patients with microsatellite stable (MSS) advanced colorectal cancer	NCT03642067	2	A/B) Nivolumab + relatlimab (160 mg) (co-administered) C) Nivolumab + Relatlimab (960 mg or 480 mg) (sequential administration) C) Nivolumab + relatlimab (160 mg) (co-administration)	Recruiting	96	ORR
Study of cabozantinib and nivolumab in refractory metastatic microsatellite stable (MSS) colorectal cancer	NCT04963283	2	Cabozantinib + nivolumab	Recruiting	46	DCR
Encorafenib, cetuximab, and nivolumab in treating patients with microsatellite stable, BRAFV600E mutated unresectable or metastatic colorectal cancer^51^	NCT04017650	1/2	Encorafenib + cetuximab + nivolumab	Recruiting	38	ORR Grade 3 TRAEs
A Study of BMS-986340 as Monotherapy and in Combination With Nivolumab or Docetaxel in Participants With Advanced Solid Tumors	NCT04895709	1/2	1A)BMS-986340 Dose Escalation 1B)BMS-986340 + Nivolumab Dose Escalation 1C)BMS-986340 + Docetaxel Dose Escalation 2A)BMS-986340 Dose Expansion 2B)BMS-986340 + Nivolumab Dose Expansion	Recruiting	665	Incidence of AEs Incidence of serious AEs Incidence of AEs meeting protocol defined DLT criteria Incidence of AEs leading to discontinuation Incidence of AEs leading to death
A study to assess efficacy of RXC004 ± nivolumab in ring finger protein 43 (RNF43) or R-spondin (RSPO) aberrated, metastatic, microsatellite stable, colorectal cancer after progression on the standard of care (SOC)^73^	NCT04907539	2	RXC004 + denosumab RXC004 + denosumab + nivolumab (opportunity to cross over to nivolumab arm at disease progression)	Recruiting	50	DCR (RXC004) ORR (RXC004 and nivolumab combination)
Avelumab combined with cetuximab and irinotecan for treatment-refractory metastatic colorectal microsatellite stable cancer (AVETUXIRI)^74^	NCT03608046	2	Avelumab + cetuximab + irinotecan	Recruiting	59	ORR
Study of PI3Kinase inhibition (copanlisib) and anti-PD-1 antibody nivolumab in relapsed/refractory solid tumors with expansions in mismatch-repair proficient (MSS) colorectal cancer^75^	NCT03711058	1/2	1) Copanlisib and nivolumab (de-escalation) 2) Copanlisib and nivolumab	Active, not recruiting	54	Determine MTD of copanlisib with fixed dose nivolumab ORR at 6 months
Regorafenib, ipilimumab and nivolumab for the treatment of chemotherapy-resistant microsatellite stable metastatic colorectal cancer^76^	NCT04362839	1	Regorafenib + Nivolumab+ Ipilimumab	Active, not recruiting	32 (39)	Recommended dose level of the combination
SX-682 and nivolumab for the treatment of RAS-mutated, MSS unresectable or metastatic colorectal cancer, the STOPTRAFFIC-1 trial^77^	NCT04599140	1/2	SX-682 (CXCR1/2 inhibitor) SX-682 + nivolumab	Recruiting	53	Incidence of AEs
Testing the addition of nivolumab to standard treatment for patients with metastatic or unresectable colorectal cancer that have a BRAF mutation^78^	NCT05308446	2	Cetuximab + encorafenib + nivolumab cetuximab + encorafenib	Recruiting	84	PFS
Nivolumab and metformin in patients with treatment-refractory MSS colorectal cancer^79^	NCT03800602	2	Nivolumab + Metformin	Active, not recruiting	24	ORR
METIMMOX-2: metastatic pMMR/MSS colorectal cancer—shaping anti-tumor immunity by oxaliplatin (METIMMOX-2)	NCT05504252	2	Nordic FLOX regimen + nivolumab	Recruiting	80	PFS
Preoperative nivolumab plus bevacizumab combined with chemotherapy before surgery in patients with pMMR/MSS colorectal cancer liver metastases	NCT05588297	2	Nivolumab + bevacizumab + CAPOX	Not yet recruiting	12	R0 recession rate PCR rate Tumor regression grade
Study to evaluate efficacy and safety of selective internal radiation therapy plus Xelox, bevacizumab and atezolizumab (immune checkpoint inhibitor) in patients with liver-dominant metastatic colorectal cancer (SIRTCI)	NCT04659382	2	XELOX + Bevacizumab + Atezolizumab + SIRT (Therasphere)	Recruiting	52	PFS at 9 months
Pixatimod (PG545) plus nivolumab in PD-1 relapsed/refractory metastatic melanoma and NSCLC and with nivolumab and low-dose cyclophosphamide in MSS metastatic colorectal carcinoma (mCRC)	NCT05061017	2	Pixatimod (PG545) + nivolumab pixatimod (PG545) + nivolumab + cyclophosphamide	Recruiting	61	ORR
Exploratory study on combined conversion immunotherapy for liver metastasis of MSS type initial unresectable colorectal cancer based on gene status	NCT05409417	2/3	Tislelizumab + XELOX + bevacizumab OR FOLFOX + cetuximab	Recruiting	40	Conversion rates R0 resection rate Drug safety
mFOLFOX6 + bevacizumab + PD-1 monoclonal antibody in local advanced MSS CRC (BASKETⅡ)	NCT04895137	2	mFOLFOX6 + Bevacizumab+ PD-1 monoclonal antibody	Recruiting	42	PCR rate
RP2/RP3 in combination with atezolizumab and bevacizumab for the treatment of patients with CRC^80^	NCT05733611	2	RP2 (genetically modified herpes simplex type 1 virus) + atezolizumab + bevacizumab RP3 (genetically modified herpes simplex type 1 virus) + atezolizumab + bevacizumab	Not yet recruiting	60	ORR
Regorafenib and PD-1 antibody in combination with radiotherapy for pMMR/MSS metastatic colorectal cancer	NCT04030260	2	Regorafenib + PD-1 antibody + RT	Recruiting	28	PFS at 6-months
A combination therapy including anti-PD-1 immunotherapy in MSS rectal cancer with resectable distal metastasis (miracle-1)	NCT05359393	2	Preoperative short-course RT + Tislelizumab + CAPEOX	Not yet recruiting	52	Tumor regression grade
Botensilimab, balstilimab and regorafenib for the treatment of patients with microsatellite stable metastatic colorectal cancer who have progressed on prior chemotherapy	NCT05672316	1/2	Botensilimab + balstilimab + regorafenib	Not yet recruiting	63	Recommended dose Incidence of DLTs ORR
A vaccine (polyPEPI1018 vaccine) and TAS-102 for the treatment of metastatic colorectal cancer^81^	NCT05130060	1	PolyPEPI1018 + TAS-102	Active, not recruiting	15	Incidence of AEs
Niraparib and panitumumab in patients with advanced or metastatic colorectal cancer (NIPAVect)^82^	NCT03983993	2	Niraparib + panitumumab	Active, not recruiting	40	Clinical benefit rate
Sintilimab (IBI308) combined with bevacizumab + XELOX regimen in metastatic colorectal cancer	NCT04194359	2	Sintilimab (IBI308) + bevacizumab + XELOX	Active, not recruiting	25	ORR TRAEs and serious AEs
CGX1321 in Subjects With Advanced Solid Tumors and CGX1321 With Pembrolizumab or Encorafenib + Cetuximab in Subjects With Advanced GI Tumors (Keynote 596)^83^	NCT02675946	1	CGX1321 CGX1321 + Pembrolizumab CGX1321 + Encorafenib + Cetuximab	Recruiting	72	Incidence of AEs
Study of NIS793 and other novel investigational combinations with SOC anti-cancer therapy for the 2L treatment of mCRC^84^	NCT04952753	2	NIS793 + SOC (mFOLFOX6/FOLFIRI + bevacizumab) NIS793 + Tislelizumab + SOC	Recruiting	268	DLTs PFS
Study of lenvatinib (MK-7902/E7080) in combination with pembrolizumab (MK-3475) versus standard of care in participants with metastatic colorectal cancer (MK-7902-017/E7080-G000-325/LEAP-017)^85^	NCT04776148	3	Lenvatinib + pembrolizumab vs. SOC (regorafenib or TAS-102)	Active, not recruiting	434	OS
Study of XL092 + atezolizumab vs regorafenib in subjects with metastatic colorectal cancer (STELLAR-303)^86^	NCT05425940	3	XL092 + atezolizumab vs. regorafenib	Recruiting	600	OS

## Discussion

Patients with MSS CRC is an important target population for ICI clinical trials due to limited therapeutic options in the later lines and the high frequency of the disease.^18^ However, unlike immune hot tumors, the effectiveness of ICI monotherapy in MSS CRC was found to be limited in previous clinical trials, particularly late in the course of the disease. So far, no studies have reported a response rate exceeding 10%.^87^ While the response to ICI therapy is limited for the majority of MSS CRC, hypermutated tumors with POLE/POLD1 mutations^88^ could represent a subgroup of MSS patients who may benefit from ICI-based therapies. Therefore, it would offer limited benefit to pursuing further ICI monotherapy in the MSS CRC in biomarker unselected cohorts, while better biomarker selection and translational research should be pursued in patients with a demonstrated response to ICI monotherapy.

In contrast to limited efficacy with ICI monotherapy and ICI-ICI combinations, the combinations with anti-VEGF agents have revealed varying results in several studies (Table 2). The interest in this area was amplified after the demonstration of a 36% ORR in the RegoNivo study^26^; however, the same success could not be reproducible in a similar study conducted in the US.^27^ Furthermore, the recent press release from the phase III LEAP-017 trial comparing pembrolizumab plus lenvatinib versus standard of care in previously treated CRC reported no statistically significant OS benefit.^85,89^ Nonetheless, the CAMILLA trial with cabozantinib plus durvalumab reported a 30% ORR^30^ and the STELLAR-303 study, a phase III randomized trial investigating XL092 and atezolizumab combination versus regorafenib is currently enrolling based on promising responses seen in the CAMILLA trial (NCT05425940).^86^

The VEGF pathway is instrumental in creating and perpetuating the immunosuppression in TME. The VEGF overexpression by tumor-associated macrophages leads to increased TGF-β secretion, activated epithelial-mesenchymal transition, and impaired dendritic maturation. These factors could contribute the immune exhaustion in the TME leading to decreased ICI efficacy.^90-92^ Therefore, combining the anti-VEGF agents and ICIs to create a synergistic immune activation emerged as a tool to improve ICI efficacy.^93^ These combinations significantly improved the survivals and became among the standard of care options in the treatment of RCC,^94^ and cervix cancer.^95^ It is important to note that although the VEGF pathway contributes significantly to the development and maintenance of immunosuppression in the tumor microenvironment (TME) similar to these cancers, it is relatively less active in CRC compared to RCC,^96^ a tumor in which ICI plus anti-VEGF TKI combination became the standard of care.

The intracrine VEGF signaling demonstrated to factor in migration and invasion in CRC cell lines and the VEGF depletion leads to significant decreases in EGFR, cMET, and phosphorylated focal adhesion kinase, critical factors in invasion and motility.^91^ Furthermore, the increased VEGF levels were correlated with hepatic metastases^97,^ and the suppression of VEGF attenuates the STAT3 phosphorylation, proliferation, and metastasis.^98^ The hepatic metastases are the most frequent sites of metastasis in patients with metastatic CRC and were linked to ICI resistance for patients with MSS CRC in several trials.^26,31^This indicates that there may be additional biological characteristics of hepatic metastases beyond increased VEGF signaling impacting immune response and abrogating antitumor immunity. Considering these points, targeting the VEGF pathway in addition to ICIs has a strong biological rationale for patients without liver metastasis and more translational research is needed to better understand the biological underpinnings of ICI resistance to deliver more effective approaches to clinical trials. However, the reason for variable efficacy across different anti-VEGF agents and across different populations is yet to be defined. Whether the cMET inhibition of cabozantinib in addition to VEGF blockade^99^ could be the reason for higher ORR in CAMILIA study^30^ should be further investigated considering the instrumental role of cMET in mediating VEGF effects in TME.

The combination of ICIs with chemotherapy is another intriguing revenue to explore ICI potential in MSS CRC. The ICI plus chemotherapy combinations created significant success in NSCLC,^100^ gastric cancer,^101^ and triple-negative breast cancer.^102^ However, a similar success could not be replicated in patients with CRC in most studies with 5-FU-based therapy and ICIs,^39,103^ and the trials with these combinations in the later lines were ceased. In contrast, there is a recent interest in the first-line trials. Oxaliplatin was previously demonstrated to increase the calreticulin and high-mobility group box 1 protein (HMGB1) levels in the murine and human CRC cell lines and corresponding immunogenic cell death (ICD) via apoptosis activation.^104^ Furthermore, oxaliplatin resulted in ICD, activation in dendritic cells and cytotoxic T cells in a murine lung carcinoma model.^105^ These immune effects could be amplified by blocking the VEGF pathway via bevacizumab.^106,107^ Therefore, recent studies tested the benefit of ICIs with 5-FU + oxaliplatin and bevacizumab backbones. However, the Checkmate 9 × 8 study, comparing FOLFOX plus bevacizumab with or without nivolumab was negative for PFS, while overall survival outcomes were more favorable for patients who received nivolumab.^44^ In contrast, a modest improvement in PFS was present with addition of atezolizumab to FOLFIRINOX plus bevacizumab in AtezoTRIBE study.^36^ Both the short follow-up times and the lack of precise and well-validated biomarkers for both studies reduced the study results’ generalization and pointed out a need for further research on the role of chemotherapy plus ICI combinations in MSS CRC. However, recently published biomarker analyses from the AtezoTRIBE study demonstrated that higher immunoscore values were associated with greater immunotherapy benefits in patients with pMMR CRC.^108,109^ If similar results are replicated in future clinical trials, the immunoscore could be a biomarker for the use of ICIs in pMMR CRC.

Patients with BRAF-mutated MSS CRC have very poor prognosis despite the recent advances with BRAF inhibitors in second-line therapy.^110,111^ The activation of the BRAF-MEK-ERK pathway leads to proliferation, survival, and immune escape with widespread effects on the TME, including increased levels of IL-6, IL-10, and VEGF, impaired dendritic cell and T-lymphocyte maturation and increased expression of immunosuppressive immune checkpoints.^112,113^ Considering that most of these effects could be reversed by ICIs, combining BRAF/MEK inhibitors with ICIs has significant potential in MSS CRC, similar to melanoma. The combination of encorafenib and cetuximab with nivolumab leads to an ORR of 45% in patients with MSS,^51^ BRAFV600E metastatic CRC in a phase I/II trial leading to a randomized phase II.^51,78^ The same success was not achieved in 2 studies with atezolizumab plus cobimetinib, an MEK inhibitor in an unselected metastatic MSS CRC population.^45,67^

In addition to further advances in the ICI-VEGF, ICI-Chemotherapy, and ICI plus BRAF inhibitor, there are several other intriguing combinations based on combinations of immune modulators like CAR-T therapies^69^ and immune activators^45^ and ICI-ICI combinations.^23,24^ The combination of a next-generation anti-CTLA4 inhibitor, botensilimab, and balstilimab (anti-PD-1) was recently evaluated in 41 patients with heavily pretreated (median of 4 prior treatments) patients with MSS CRC. The ORR and DCR were 24% and 73%, respectively. Additionally, in patients without an active liver metastasis the ORR and DCR was 42% and 96% indicating the benefit is perhaps again limited to patients without active liver metastasis.^23^ Notably, in this study, no patient with active liver metastasis has responded to this combination. Half of the responding patients had RAS mutations, and only one responding had a high tumor mutational burden. Additionally, the POLE mutation was not present in 10 patients with a response to therapy. No grades 4-5 toxicities were observed.^23^

Another unmet need for research is the combination of anti-TGF-β inhibitors with ICIs. The TGF-β is the main driver of epithelial-to-mesenchymal (EMT) transition and a potent proliferative and pro-invasive signal in TME. Additionally, TGF-β has pivotal effects on immune surveillance. The TGF-β may have inhibitory effects on T-helper1 phenotype differentiation, CD8 T-cell activity, IL-2 expression, and natural cell killer proliferation and function and increased the percentage of immunosuppressive M2 macrophages and myeloid-derived suppressor cells (MDSC).^114^ The TGF-β pathway is active starting from the earlier phases of CRC carcinogenesis and is even more prominent in the CRC hepatic metastases. Due to these effects on the immune system and the association between TGF-beta and resistance to ICIs, combining TGF-β inhibitors with ICIs emerged as a feasible strategy to activate immune machinery.^115^ In a recent phase II open-label trial, the combination of vactosertib (selective TGF-β receptor I kinase inhibitor) and pembrolizumab led to an ORR of 15.2% in patients with MSS CRC who had progression under all available options. The combination was safe with grade 3 or higher treatment-related adverse events in 3 of 33 patients.^71^ While these results hold promise for the future, it should be noted that 82% of the patients had consensus molecular subtype (CMS) 4 disease,^71^ a subtype in which TGF-β pathway alterations are pivotal.^116,117^ Further exploration of TGF-β and ICI combinations is needed and could be especially important for patients with CMS4 disease. Furthermore, patient stratification according to CMS molecular subtypes could be of benefit for treatment individualization and improvement of outcomes. Several other TME modifiers, like maraviroc, and ^72^ pixatimod were also combined with ICIs, although the success was limited, and no further studies are planned with these agents.^56^

There are several limitations in the systematic review, mostly inherent to the included studies. Most of the studies included had very limited sample sizes and included heterogeneous populations regarding the treatment line and previous treatments. The available studies mostly used regorafenib as the standard third-line treatment, although the current standard treatment is different in this setting. Additionally, detailed subgroup analyses were absent in most studies, limiting the ability to pool the data regarding possible subgroups with a higher benefit with ICIs. For example, patients without liver metastases seem to benefit more from ICIs; however, reporting for this subgroup of patients was not available in most papers.

## Conclusion

In conclusion, in the systematic review of ICI studies in MSS CRC, we observed that the ICI monotherapy was significantly limited in efficacy, while the combinations with anti-VEGF TKI, other immune-modulatory agents and BRAF inhibitors may have more efficacy. However, with varying results seen in phase II studies with TKI and ICI therapy combinations with a recently negative phase III trial of LEAP-17, the benefit of this combination in MSS CRC appears to be limited. The data regarding the chemotherapy plus ICI combinations in the first-line treatments reported modest benefits. Further research is needed to delineate the best strategies and best combinations to implement ICIs in the treatment of patients with MSS CRC.

## Data Availability

No new data were generated or analyzed in support of this research.

## References

[1] Baselga J , Bhardwaj N, Cantley LC, et al. AACR cancer progress report 2015. Clin Cancer Res: Off J Am Assoc Cancer Res. 2015;21(19 Suppl):S1-128. 10.1158/1078-0432.CCR-15-1846 PMC5001568 26429991

[2] Ribas A. Releasing the brakes on cancer immunotherapy. N Engl J Med. 2015;373(16):1490-1492. 10.1056/NEJMp1510079 26348216

[3] Darvin P , Toor SM, Sasidharan Nair V, Elkord E. Immune checkpoint inhibitors: recent progress and potential biomarkers. Exp Mol Med. 2018;50(12):1-11. 10.1038/s12276-018-0191-1 PMC6292890 30546008

[4] Wolchok JD , Chiarion-Sileni V, Gonzalez R, et al. Long-term outcomes with nivolumab plus ipilimumab or nivolumab alone versus ipilimumab in patients with advanced melanoma. J Clin Oncol. 2021;40(2):127-137. 10.1200/JCO.21.02229 34818112 PMC8718224

[5] Reck M , Rodríguez-Abreu D, Robinson AG, et al. Updated analysis of KEYNOTE-024: pembrolizumab versus platinum-based chemotherapy for advanced non-small-cell lung cancer with PD-L1 tumor proportion score of 50% or greater. J Clin Oncol. 2019;37(7):537-546. 10.1200/JCO.18.00149 30620668

[6] Motzer RJ , Powles T, Burotto M, et al. Nivolumab plus cabozantinib versus sunitinib in first-line treatment for advanced renal cell carcinoma (CheckMate 9ER): long-term follow-up results from an open-label, randomised, phase 3 trial. Lancet Oncol. 2022;23(7):888-898. 10.1016/S1470-2045(22)00290-X 35688173 PMC10305087

[7] Maio M , Ascierto PA, Manzyuk L, et al. Pembrolizumab in microsatellite instability high or mismatch repair deficient cancers: updated analysis from the phase II KEYNOTE-158 study. Ann Oncol 2022;33(9):929-938. 10.1016/j.annonc.2022.05.519 35680043

[8] Marabelle A , Fakih M, Lopez J, et al. Association of tumour mutational burden with outcomes in patients with advanced solid tumours treated with pembrolizumab: prospective biomarker analysis of the multicohort, open-label, phase 2 KEYNOTE-158 study. Lancet Oncol. 2020;21(10):1353-1365. 10.1016/S1470-2045(20)30445-9 32919526

[9] Yarchoan M , Hopkins A, Jaffee EM. Tumor mutational burden and response rate to PD-1 inhibition. N Engl J Med. 2017;377(25):2500-2501. 10.1056/NEJMc1713444 29262275 PMC6549688

[10] Hegde PS , Chen DS. Top 10 challenges in cancer immunotherapy. Immunity. 2020;52(1):17-35. 10.1016/j.immuni.2019.12.011 31940268

[11] Le DT , Uram JN, Wang H, et al. PD-1 Blockade in tumors with mismatch-repair deficiency. N Engl J Med. 2015;372(26):2509-2520. 10.1056/NEJMoa1500596 26028255 PMC4481136

[12] Lenz HJ , Van Cutsem E, Luisa Limon M, et al. First-line nivolumab plus low-dose ipilimumab for microsatellite instability-high/mismatch repair-deficient metastatic colorectal cancer: the phase II CheckMate 142 study. J Clin Oncol. 2022;40(2):161-170. 10.1200/JCO.21.01015 34637336

[13] Diaz LA Jr , Shiu K-K, Kim T-W, et al; KEYNOTE-177 Investigators. Pembrolizumab versus chemotherapy for microsatellite instability-high or mismatch repair-deficient metastatic colorectal cancer (KEYNOTE-177): final analysis of a randomised, open-label, phase 3 study. Lancet Oncol. 2022;23(5):659-670. 10.1016/S1470-2045(22)00197-8 35427471 PMC9533375

[14] Fabrizio DA , George TJ r, Dunne RF, et al. Beyond microsatellite testing: assessment of tumor mutational burden identifies subsets of colorectal cancer who may respond to immune checkpoint inhibition. J Gastrointest Oncol. 2018;9(4):610-617. 10.21037/jgo.2018.05.06 30151257 PMC6087857

[15] Overman MJ , Kopetz S, McDermott RS, et al. Nivolumab ± ipilimumab in treatment (tx) of patients (pts) with metastatic colorectal cancer (mCRC) with and without high microsatellite instability (MSI-H): CheckMate-142 interim results. J Clin Oncol. 2016;34(15_suppl):3501-3501. 10.1200/jco.2016.34.15_suppl.3501

[16] Lin A , Zhang J, Luo P. Crosstalk between the MSI status and tumor microenvironment in colorectal cancer. Front Immunol. 2020;11:2039. 10.3389/fimmu.2020.02039 32903444 PMC7435056

[17] Ghiringhelli F , Fumet JD. Is There a place for immunotherapy for metastatic microsatellite stable colorectal cancer? Front Immunol. 2019;10:1816. 10.3389/fimmu.2019.01816 31447840 PMC6691024

[18] Weng J , Li S, Zhu Z, et al. Exploring immunotherapy in colorectal cancer. J Hematol Oncol. 2022;15(1):95. 10.1186/s13045-022-01294-4 35842707 PMC9288068

[19] Page MJ , McKenzie JE, Bossuyt PM, et al. The PRISMA 2020 statement: an updated guideline for reporting systematic reviews. BMJ 2021;372:n71. 10.1136/bmj.n71 33782057 PMC8005924

[20] Slim K , Nini E, Forestier D, et al. Methodological index for non-randomized studies (minors): development and validation of a new instrument. ANZ J Surg 2003;73(9):712-716. 10.1046/j.1445-2197.2003.02748.x 12956787

[21] Sterne JAC , Savović J, Page MJ, et al. RoB 2: a revised tool for assessing risk of bias in randomised trials. BMJ 2019;366:l4898. 10.1136/bmj.l4898 31462531

[22] Middleton G , Liu W, Savage J, et al. 426P Assessing nivolumab in class II expressing microsatellite stable (pMMR) colorectal cancer (CRC): Results of the ANICCA-Class II trial. Ann Oncol. 2022;33:S729-S730. 10.1016/j.annonc.2022.07.564

[23] Bullock A , Grossman J, Fakih M, et al. LBA O-9 Botensilimab, a novel innate/adaptive immune activator, plus balstilimab (anti-PD-1) for metastatic heavily pretreated microsatellite stable colorectal cancer. Ann Oncol. 2022;33:S376.

[24] Chen EX , Jonker DJ, Loree JM, et al. Effect of Combined immune checkpoint inhibition vs best supportive care alone in patients with advanced colorectal cancer: the Canadian cancer trials group CO.26 study. JAMA Oncol. 2020;6(6):831-838. 10.1001/jamaoncol.2020.0910 32379280 PMC7206536

[25] Wilky B , Margolin K, Sanborn RE, et al. 778 Botensilimab, a novel innate/adaptive immune activator, plus or minus balstilimab (anti-PD-1) in “cold” and IO refractory metastatic solid tumors. J ImmunoTher Cancer. 2022.

[26] Fukuoka S , Hara H, Takahashi N, et al. Regorafenib plus nivolumab in patients with advanced gastric or colorectal cancer: an open-label, dose-escalation, and dose-expansion phase Ib trial (REGONIVO, EPOC1603). J Clin Oncol: Off J Am Soc Clin Oncol. 2020;38(18):2053-2061. 10.1200/JCO.19.03296 32343640

[27] Kim R , Imanirad I, Strosberg J, Carballido E, Kim D. PD-2 Final result of phase IB study of regorafenib and nivolumab in mismatch repair proficient advanced refractory colorectal cancer. Ann Oncol. 2021;32:S199.

[28] Ma S , Chen R, Hou X, et al. Toripalimab with Fruquintinib as the third-line treatment for refractory advanced metastatic colorectal cancer: results of a single-arm, single-center, prospective, phase II clinical study. J Clin Oncol. 2022;40(16_suppl):e15573-e15573. 10.1200/jco.2022.40.16_suppl.e15573 PMC10186513 37201046

[29] Zhang W , Sun Y, Jiang Z, et al. 423P Fruquintinib plus sintilimab in refractory repair-proficient (pMMR)/microsatellite stable (MSS) metastatic colorectal cancer (mCRC): Preliminary clinical results and biomarker analyses from a phase II study. Ann Oncol. 2022;33:S728.

[30] Saeed A , Park R, Dai J, et al. Cabozantinib plus durvalumab in advanced gastroesophageal cancer and other gastrointestinal malignancies: phase Ib CAMILLA trial results. Cell Rep Med. 2023;4(2):100916. 10.1016/j.xcrm.2023.100916 36702123 PMC9975105

[31] Fakih M , Raghav KPS, Chang DZ, et al. Regorafenib plus nivolumab in patients with mismatch repair-proficient/microsatellite stable metastatic colorectal cancer: a single-arm, open-label, multicentre phase 2 study. eClinicalMedicine 2023;58:101917. 10.1016/j.eclinm.2023.101917 37090438 PMC10119887

[32] Barzi A , Azad NS, Yang Y, et al. Phase I/II study of regorafenib (rego) and pembrolizumab (pembro) in refractory microsatellite stable colorectal cancer (MSSCRC). J Clin Oncol. 2022;40(4_suppl):15-15. 10.1200/jco.2022.40.4_suppl.015

[33] Cousin S , Bellera CA, Guégan JP, et al. REGOMUNE: a phase II study of regorafenib plus avelumab in solid tumors—results of the non-MSI-H metastatic colorectal cancer (mCRC) cohort. *JCO*. 2020;38:4019-4019. 10.1200/JCO.2020.38.15_suppl.4019

[34] Gomez-Roca C , Yanez E, Im S-A, et al. LEAP-005: A phase II multicohort study of lenvatinib plus pembrolizumab in patients with previously treated selected solid tumors—results from the colorectal cancer cohort. JCO. 2021;39:94-94. 10.1200/JCO.2021.39.3_suppl.94

[35] Argilés G , André T, Hollebecque A, et al. Phase I dose-escalation of trifluridine/tipiracil in combination with oxaliplatin in patients with metastatic colorectal cancer. Eur J Cancer (Oxford, England : 1990) 2019;112:12-19. 10.1016/j.ejca.2019.01.101 30889492

[36] Antoniotti C , Rossini D, Pietrantonio F, et al; GONO Foundation Investigators. Upfront FOLFOXIRI plus bevacizumab with or without atezolizumab in the treatment of patients with metastatic colorectal cancer (AtezoTRIBE): a multicentre, open-label, randomised, controlled, phase 2 trial. Lancet Oncol. 2022;23(7):876-887. 10.1016/S1470-2045(22)00274-1 35636444

[37] Antoniotti C , Borelli B, Rossini D, et al. AtezoTRIBE: a randomised phase II study of FOLFOXIRI plus bevacizumab alone or in combination with atezolizumab as initial therapy for patients with unresectable metastatic colorectal cancer. BMC Cancer 2020;20(1):1-9. 10.1186/s12885-020-07169-6 PMC7376656 32698790

[38] Fang X , Zhong C, Weng S, et al. Sintilimab plus bevacizumab and CapeOx (BBCAPX) on first-line treatment in patients with RAS mutant, microsatellite stable, metastatic colorectal cancer: study protocol of a randomized, open-label, multicentric study. BMC Cancer 2023;23(1):676. 10.1186/s12885-023-11139-z 37464378 PMC10354966

[39] Mettu NB , Ou F-S, Zemla TJ, et al. Assessment of Capecitabine and bevacizumab with or without atezolizumab for the treatment of refractory metastatic colorectal cancer: a randomized clinical trial. JAMA Network Open 2022;5(2):e2149040-e2149040. 10.1001/jamanetworkopen.2021.49040 35179586 PMC8857687

[40] Morano F , Raimondi A, Pagani F, et al. Temozolomide followed by combination with low-dose ipilimumab and nivolumab in patients with microsatellite-stable, O(6)-methylguanine-DNA methyltransferase-silenced metastatic colorectal cancer: the MAYA trial. J Clin Oncol. 2022;40(14):1562-1573. 10.1200/JCO.21.02583 35258987 PMC9084437

[41] Patel MR , Falchook GS, Hamada K, Makris L, Bendell JC. A phase 2 trial of trifluridine/tipiracil plus nivolumab in patients with heavily pretreated microsatellite-stable metastatic colorectal cancer. Cancer Med 2021;10(4):1183-1190. 10.1002/cam4.3630 33544407 PMC7926002

[42] Taylor K , Loo Yau H, Chakravarthy A, et al. An open-label, phase II multicohort study of an oral hypomethylating agent CC-486 and durvalumab in advanced solid tumors. J ImmunoTher Cancer. 2020;8(2):e000883. 10.1136/jitc-2020-000883 32753546 PMC7406114

[43] Fumet J , Chibaudel B, Bennouna J, et al. 433P Durvalumab and tremelimumab in combination with FOLFOX in patients with previously untreated RAS-mutated metastatic colorectal cancer: First results of efficacy at one year for phase II MEDITREME trial. Ann Oncol. 2021;32:S551.

[44] Lenz H-J , Parikh AR, Spigel DR, et al. Nivolumab (NIVO)+ 5-fluorouracil/leucovorin/oxaliplatin (mFOLFOX6)/bevacizumab (BEV) versus mFOLFOX6/BEV for first-line (1L) treatment of metastatic colorectal cancer (mCRC): phase 2 results from CheckMate 9X8. JCO. 2022;40:8-8.

[45] Hellmann MD , Kim TW, Lee CB, et al. Phase Ib study of atezolizumab combined with cobimetinib in patients with solid tumors. Ann Oncol. 2019;30(7):1134-1142. 10.1093/annonc/mdz113 30918950 PMC6931236

[46] Español-Rego M , Fernández-Martos C, Elez E, et al. A Phase I-II multicenter trial with Avelumab plus autologous dendritic cell vaccine in pre-treated mismatch repair-proficient (MSS) metastatic colorectal cancer patients; GEMCAD 1602 study. Cancer Immunol, Immunother 2023;72(4):827-840. 10.1007/s00262-022-03283-5 36083313 PMC10025226

[47] Kawazoe A , Itahashi K, Yamamoto N, et al. TAS-116 (Pimitespib), an oral HSP90 inhibitor, in combination with nivolumab in patients with colorectal cancer and other solid tumors: an open-label, dose-finding, and expansion phase Ib trial (EPOC1704). Clin Cancer Res 2021;27(24):6709-6715. 10.1158/1078-0432.CCR-21-1929 34593531

[48] Kawazoe A , Kuboki Y, Shinozaki E, et al. Multicenter phase I/II trial of napabucasin and pembrolizumab in patients with metastatic colorectal cancer (EPOC1503/SCOOP trial). Clin Cancer Res 2020;26(22):5887-5894. 10.1158/1078-0432.CCR-20-1803 32694160

[49] Fakih M , Harb W, Mahadevan D, et al. Safety and efficacy of the tumor-selective adenovirus enadenotucirev, in combination with nivolumab, in patients with advanced/metastatic epithelial cancer: a phase I clinical trial (SPICE). J ImmunoTher Cancer. 2023;11(4):e006561. 10.1136/jitc-2022-006561 37094988 PMC10151977

[50] Monge C , Xie C, Myojin Y, et al. Phase I/II study of PexaVec in combination with immune checkpoint inhibition in refractory metastatic colorectal cancer. J ImmunoTher Cancer. 2023;11(2):e005640. 10.1136/jitc-2022-005640 36754451 PMC9923269

[51] Morris VK , Parseghian CM, Escano M, et al. Phase I/II trial of encorafenib, cetuximab, and nivolumab in patients with microsatellite stable, BRAF V600E metastatic colorectal cancer. JCO. 2022;40:12-12.

[52] Rahma OE , Tyan K, Giobbie-Hurder A, et al. Phase IB study of ziv-aflibercept plus pembrolizumab in patients with advanced solid tumors. J ImmunoTher Cancer. 2022;10(3):e003569. 10.1136/jitc-2021-003569 35264434 PMC8915279

[53] Suarez-Carmona M , Williams A, Schreiber J, et al. Combined inhibition of CXCL12 and PD-1 in MSS colorectal and pancreatic cancer: modulation of the microenvironment and clinical effects. J ImmunoTher Cancer. 2021;9(10):e002505. 10.1136/jitc-2021-002505 34607895 PMC8491418

[54] Bendell J , LoRusso P, Overman M, et al. First-in-human study of oleclumab, a potent, selective anti-CD73 monoclonal antibody, alone or in combination with durvalumab in patients with advanced solid tumors. Cancer Immunol, Immunother 2023;72(7):2443-2458. 10.1007/s00262-023-03430-6 37016126 PMC10264501

[55] Bassetti MF , Turk AA, Lubner SJ, et al. A phase Ib study of pembrolizumab (Pem) in combination with stereotactic body radiotherapy (SBRT) for resectable liver metastatic MSS colorectal cancer (CRC): a postoperative safety analysis. J Clin Oncol. 2019;37(15_suppl):e15047-e15047. 10.1200/jco.2019.37.15_suppl.e15047

[56] Dredge K , Brennan TV, Hammond E, et al. A phase I study of the novel immunomodulatory agent PG545 (pixatimod) in subjects with advanced solid tumours. Br J Cancer. 2018;118(8):1035-1041. 10.1038/s41416-018-0006-0 29531325 PMC5931096

[57] Johnson B , Haymaker CL, Parra ER, et al. Phase II study of durvalumab (anti-PD-L1) and trametinib (MEKi) in microsatellite stable (MSS) metastatic colorectal cancer (mCRC). J ImmunoTher Cancer. 2022;10(8):e005332. 10.1136/jitc-2022-005332 36007963 PMC9422817

[58] Naing A , Fan J, Lee BH, et al. Safety, pharmacokinetics, pharmacodynamics profiles and preliminary antitumor activity of phase 1b/2a study of NT-I7, a long-acting interleukin-7, plus pembrolizumab in patients with advanced solid tumors: the phase 1b data report. J Clin Oncol. 2021;39(15_suppl):2594-2594. 10.1200/jco.2021.39.15_suppl.259434019431

[59] Lee JJ , Yothers G, George TJ, et al. NSABP FC-9: Phase II study of dual immune checkpoint blockade (ICB) with durvalumab (D) plus tremelimumab (T) following palliative hypofractionated radiotherapy (SBRT) in patients (pts) with microsatellite-stable (MSS) metastatic colorectal cancer (mCRC) progressing on chemotherapy. J Clin Oncol. 2018;36(15_suppl):e15681-e15681. 10.1200/jco.2018.36.15_suppl.e15681

[60] Lee MS , Loehrer PJ, Imanirad I, et al. Phase II study of ipilimumab, nivolumab, and panitumumab in patients with KRAS/NRAS/BRAF wild-type (WT) microsatellite stable (MSS) metastatic colorectal cancer (mCRC). J Clin Oncol. 2021;39(3_suppl):7-7. 10.1200/jco.2021.39.3_suppl.733275489

[61] Friedrich T , Blatchford PJ, Lentz RW, et al. A phase II study of pembrolizumab, binimetinib, and bevacizumab in patients with microsatellite-stable, refractory, metastatic colorectal cancer (mCRC). J Clin Oncol. 2022;40(4_suppl):118-118. 10.1200/jco.2022.40.4_suppl.11834855471

[62] Parikh AR , Szabolcs A, Allen JN, et al. Radiation therapy enhances immunotherapy response in microsatellite stable colorectal and pancreatic adenocarcinoma in a phase II trial. *Nat Cancer.* 2021;2(11):1124-1135. 10.1038/s43018-021-00269-7 35122060 PMC8809884

[63] Redman JM , Tsai Y-T, Weinberg BA, et al. A randomized phase II trial of mFOLFOX6 + bevacizumab alone or with AdCEA vaccine + avelumab immunotherapy for untreated metastatic colorectal cancer. Oncologist 2022;27(3):198-209. 10.1093/oncolo/oyab046 35274710 PMC8914498

[64] Saunders MP , Graham J, Cunningham D, et al. CXD101 and nivolumab in patients with metastatic microsatellite-stable colorectal cancer (CAROSELL): a multicentre, open-label, single-arm, phase II trial. ESMO Open 2022;7(6):100594. 10.1016/j.esmoop.2022.100594 36327756 PMC9808483

[65] Wang C , Park J, Ouyang C, et al. A pilot feasibility study of yttrium-90 liver radioembolization followed by durvalumab and tremelimumab in patients with microsatellite stable colorectal cancer liver metastases. Oncologist 2020;25(5):382-e776. 10.1634/theoncologist.2019-0924 31857446 PMC7216435

[66] Yang J , Zhou W, Ma Y, et al. The response of PD-1 inhibitor combined with radiotherapy and GM-CSF(PRaG) with or without IL-2 in microsatellite stable metastatic colorectal cancer: analysis of pooled data from two phase II trials. J Clin Oncol. 2022;40(16_suppl):e15561-e15561. 10.1200/jco.2022.40.16_suppl.e15561

[67] Eng C , Kim TW, Bendell J, et al; IMblaze370 Investigators. Atezolizumab with or without cobimetinib versus regorafenib in previously treated metastatic colorectal cancer (IMblaze370): a multicentre, open-label, phase 3, randomised, controlled trial. Lancet Oncol. 2019;20(6):849-861. 10.1016/S1470-2045(19)30027-0 31003911

[68] Monjazeb AM , Giobbie-Hurder A, Lako A, et al. A randomized trial of combined PD-L1 and CTLA-4 inhibition with targeted low-dose or hypofractionated radiation for patients with metastatic colorectal cancer. Clin Cancer Res. 2021;27(9):2470-2480. 10.1158/1078-0432.CCR-20-4632 33568343 PMC8102320

[69] Zhang C , Wang Z, Yang Z, et al. Phase I escalating-dose trial of CAR-T therapy targeting CEA(+) metastatic colorectal cancers. Mol Ther. 2017;25(5):1248-1258. 10.1016/j.ymthe.2017.03.010 28366766 PMC5417843

[70] Martinelli E , Martini G, Famiglietti V, et al. Cetuximab rechallenge plus avelumab in pretreated patients with RAS wild-type metastatic colorectal cancer: the phase 2 single-arm clinical CAVE trial. JAMA Oncol. 2021;7(10):1529-1535. 10.1001/jamaoncol.2021.2915 34382998 PMC8531995

[71] Kim TW , Lee KW, Ahn JB, et al. Efficacy and safety of vactosertib and pembrolizumab combination in patients with previously treated microsatellite stable metastatic colorectal cancer. J Clin Oncol. 2021;39(15_suppl):3573-3573.

[72] Haag GM , Springfeld C, Grün B, et al. Pembrolizumab and maraviroc in refractory mismatch repair proficient/microsatellite-stable metastatic colorectal cancer – the PICCASSO phase I trial. Eur J Cancer. 2022;167:112-122. 10.1016/j.ejca.2022.03.017 35427833

[73] Kopetz S , Morris VK, O’Neil B, et al. A multi-arm, phase 2, open-label study to assess the efficacy of RXC004 as monotherapy and in combination with nivolumab in patients with ring finger protein 43 (RNF43) or R-spondin (RSPO) aberrated, metastatic, microsatellite stable colorectal cancer following standard treatments. J Clin Oncol. 2022;40(16_suppl):TPS3637-TPS3637. 10.1200/jco.2022.40.16_suppl.tps3637

[74] Huyghe N , Cuyper AD, Sinapi I, et al. Interim analysis of the phase II AVETUXIRI trial: avelumab combined with cetuximab and irinotecan for treatment of refractory microsatellite stable (MSS) metastatic colorectal cancer (mCRC). J Clin Oncol. 2022;40(16_suppl):3595-3595.

[75] Jakubowski C , Collins NB, Sugar EA, et al. A phase I/II study of PI3Kinase inhibition with copanlisib combined with the anti-PD-1 antibody nivolumab in relapsed/refractory solid tumors with expansions in MSS colorectal cancer. J Clin Oncol. 2020;38(15_suppl):TPS4114-TPS4114. 10.1200/jco.2020.38.15_suppl.tps4114

[76] Fakih M , Sandhu J, Lim D, et al. Regorafenib, ipilimumab, and nivolumab for patients with microsatellite stable colorectal cancer and disease progression with prior chemotherapy: a phase 1 nonrandomized clinical trial. JAMA Oncol. 2023;9(5):627-634. 10.1001/jamaoncol.2022.7845 36892833 PMC9999273

[77] Johnson B , Kopetz S, Hwang H, et al. STOPTRAFFIC-1: A phase I/II trial of SX-682 in combination with nivolumab for refractory RAS-mutated microsatellite stable (MSS) metastatic colorectal cancer (mCRC). J Clin Oncol. 2022;40(16_suppl):TPS3638-TPS3638. 10.1200/jco.2022.40.16_suppl.tps3638

[78] Morris VK , Guthrie KA, Kopetz S, et al. Randomized phase II trial of encorafenib and cetuximab with or without nivolumab for patients with previously treated, microsatellite stable, BRAFV600E metastatic and/or unresectable colorectal cancer: SWOG S2107. J Clin Oncol. 2023;41(4_suppl):TPS265-TPS265.

[79] Akce M , Rupji M, Switchenko JM, et al. Phase II trial of nivolumab and metformin in patients with treatment refractory microsatellite stable metastatic colorectal cancer. J Clin Oncol. 2021;39(3_suppl):95-95. 10.1200/jco.2021.39.3_suppl.95 37852737 PMC10603338

[80] Shepard DR , Ahmed M, Bekaii-Saab TS, Wolff J. An open-label clinical trial of RP2 and RP3 oncolytic immunotherapy in combination with atezolizumab and bevacizumab for the treatment of patients with advanced colorectal carcinoma. J Clin Oncol. 2023;41(16_suppl):TPS3628-TPS3628. 10.1200/jco.2023.41.16_suppl.tps3628

[81] Hubbard JM , Zemla TJ, Graham RP, et al. Phase Ib open-label study to evaluate safety, tolerability, immunogenicity, and efficacy of multiple subcutaneous injections of PolyPEPI1018 vaccine as an add-on immunotherapy to TAS-102 in participants with late-stage microsatellite-stable metastatic colorectal cancer (MSS mCRC; OBERTO-201). J Clin Oncol. 2023;41(4_suppl):147-147. 10.1200/jco.2023.41.4_suppl.147

[82] Alese OB , Diab M, Gbolahan OB, et al. Nipavect: phase II study of niraparib and panitumumab in advanced RAS WT colorectal cancer. J Clin Oncol. 2023;41(16_suppl):3579-3579. 10.1200/jco.2023.41.16_suppl.3579

[83] Giannakis M , Le DT, Pishvaian MJ, et al. Phase 1 study of WNT pathway porcupine inhibitor CGX1321 and phase 1b study of CGX1321 + pembrolizumab (pembro) in patients (pts) with advanced gastrointestinal (GI) tumors. J Clin Oncol. 2023;41(16_suppl):3514-3514. 10.1200/jco.2023.41.16_suppl.3514

[84] Segal N , Rivera F, Tournigand C, et al. P-23 Phase II study (daNIS-3) of the anti–TGF-β monoclonal antibody NIS793 and other new investigational drug combinations with standard-of-care therapy vs standard-of-care alone in patients with second-line metastatic colorectal cancer. Ann Oncol. 2022;33:S254-S255. 10.1016/j.annonc.2022.04.114

[85] Yoshino T , Fu R, Hawk N, et al. 506TiP pembrolizumab plus lenvatinib versus standard of care for previously treated metastatic colorectal cancer (mCRC): phase III LEAP-017 study. Ann Oncol. 2021;32:S580.

[86] Hecht JR , Tabernero J, Parikh AR, et al. STELLAR-303: a phase 3 study of XL092 in combination with atezolizumab versus regorafenib in patients with previously treated metastatic colorectal cancer (mCRC). J Clin Oncol. 2023;41(4_suppl):TPS267-TPS267. 10.1200/jco.2023.41.4_suppl.tps267

[87] Sahin IH , Ciombor KK, Diaz LA, Yu J, Kim R. Immunotherapy for microsatellite stable colorectal cancers: challenges and novel therapeutic avenues. Am Soc Clin Oncol Educational Book 2022;42:1-12. 10.1200/edbk_349811 35658496

[88] Hu H , Cai W, Wu D, et al. Ultra-mutated colorectal cancer patients with POLE driver mutations exhibit distinct clinical patterns. Cancer Med 2021;10(1):135-142. 10.1002/cam4.3579 33125191 PMC7826451

[89] Kawazoe A , Xu R, Passhak M, et al. LBA-5 lenvatinib plus pembrolizumab versus standard of care for previously treated metastatic colorectal cancer (mCRC): the phase 3 LEAP-017 study. Ann Oncol. 2023;34:S179. 10.1200/JCO.23.02736 PMC11328923 38833658

[90] Lugano R , Ramachandran M, Dimberg A. Tumor angiogenesis: causes, consequences, challenges and opportunities. Cell Mol Life Sci: CMLS 2020;77(9):1745-1770. 10.1007/s00018-019-03351-7 31690961 PMC7190605

[91] Bhattacharya R , Fan F, Wang R, et al. Intracrine VEGF signalling mediates colorectal cancer cell migration and invasion. Br J Cancer. 2017;117(6):848-855. 10.1038/bjc.2017.238 28742793 PMC5589988

[92] Min AKT , Mimura K, Nakajima S, et al. Therapeutic potential of anti-VEGF receptor 2 therapy targeting for M2-tumor-associated macrophages in colorectal cancer. Cancer Immunol, Immunother : CII 2021;70(2):289-298. 10.1007/s00262-020-02676-8 32705303 PMC10991089

[93] Hack SP , Zhu AX, Wang Y. Augmenting anticancer immunity through combined targeting of angiogenic and PD-1/PD-L1 pathways: challenges and opportunities. Front Immunol. 2020;11:598877. 10.3389/fimmu.2020.598877 33250900 PMC7674951

[94] Powles T , Plimack ER, Soulières D, et al. Pembrolizumab plus axitinib versus sunitinib monotherapy as first-line treatment of advanced renal cell carcinoma (KEYNOTE-426): extended follow-up from a randomised, open-label, phase 3 trial. Lancet Oncol. 2020;21(12):1563-1573. 10.1016/S1470-2045(20)30436-8 33284113

[95] Colombo N , Dubot C, Lorusso D, et al; KEYNOTE-826 Investigators. Pembrolizumab for persistent, recurrent, or metastatic cervical cancer. N Engl J Med. 2021;385(20):1856-1867. 10.1056/NEJMoa2112435 34534429

[96] Zhang W , Vallboehmer D, Mizutomo A, et al. Differential gene expression levels of vascular endothelial growth factor (VEGF) and its receptors in renal cell cancer and colorectal cancer patients. Cancer Res. 2006;66(8_Supplement):1060-1060.

[97] Konno H , Abe J, Kaneko T, et al. Urokinase receptor and vascular endothelial growth factor are synergistically associated with the liver metastasis of colorectal cancer. Jpn J Cancer Res. 2001;92(5):516-523. 10.1111/j.1349-7006.2001.tb01124.x 11376560 PMC5926736

[98] Chen SH , Murphy DA, Lassoued W, et al. Activated STAT3 is a mediator and biomarker of VEGF endothelial activation. Cancer Biol Ther. 2008;7(12):1994-2003. 10.4161/cbt.7.12.6967 18981713 PMC2932444

[99] Yakes FM , Chen J, Tan J, et al. Cabozantinib (XL184), a novel MET and VEGFR2 inhibitor, simultaneously suppresses metastasis, angiogenesis, and tumor growth. Mol Cancer Ther. 2011;10(12):2298-2308. 10.1158/1535-7163.MCT-11-0264 21926191

[100] Novello S , Kowalski DM, Luft A, et al. Pembrolizumab Plus chemotherapy in squamous non–small-cell lung cancer: 5-year update of the phase III KEYNOTE-407 study. J Clin Oncol. 2023;41(11):1999-2006. 10.1200/jco.22.01990 36735893 PMC10082300

[101] Janjigian YY , Shitara K, Moehler M, et al. First-line nivolumab plus chemotherapy versus chemotherapy alone for advanced gastric, gastro-oesophageal junction, and oesophageal adenocarcinoma (CheckMate 649): a randomised, open-label, phase 3 trial. Lancet (London, England) 2021;398(10294):27-40. 10.1016/S0140-6736(21)00797-2 34102137 PMC8436782

[102] Cortes J , Rugo HS, Cescon DW, et al; KEYNOTE-355 Investigators. Pembrolizumab plus chemotherapy in advanced triple-negative breast cancer. N Engl J Med. 2022;387(3):217-226. 10.1056/NEJMoa2202809 35857659

[103] Mettu NB , Twohy E, Ou FS, et al. BACCI: a phase II randomized, double-blind, multicenter, placebo-controlled study of capecitabine (C) bevacizumab (B) plus atezolizumab (A) or placebo (P) in refractory metastatic colorectal cancer (mCRC): an ACCRU network study. Ann Oncol. 2019;30:v203.

[104] Tesniere A , Schlemmer F, Boige V, et al. Immunogenic death of colon cancer cells treated with oxaliplatin. Oncogene. 2010;29(4):482-491. 10.1038/onc.2009.356 19881547

[105] Sun F , Cui L, Li T, et al. Oxaliplatin induces immunogenic cells death and enhances therapeutic efficacy of checkpoint inhibitor in a model of murine lung carcinoma. J Recept Signal Transduct Res. 2019;39(3):208-214. 10.1080/10799893.2019.1655050 31441696

[106] Osada T , Chong G, Tansik R, et al. The effect of anti-VEGF therapy on immature myeloid cell and dendritic cells in cancer patients. Cancer Immunol Immunother. 2008;57(8):1115-1124. 10.1007/s00262-007-0441-x 18193223 PMC4110970

[107] Saltz LB , Clarke S, Díaz-Rubio E, et al. Bevacizumab in combination with oxaliplatin-based chemotherapy as first-line therapy in metastatic colorectal cancer: a randomized phase III study. J Clin Oncol. 2008;26(12):2013-2019. 10.1200/JCO.2007.14.9930 18421054

[108] Moretto R , Rossini D, Catteau A, et al. Dissecting tumor lymphocyte infiltration to predict benefit from immune-checkpoint inhibitors in metastatic colorectal cancer: lessons from the AtezoT RIBE study. J ImmunoTher Cancer. 2023;11(4):e006633. 10.1136/jitc-2022-006633 37085190 PMC10124320

[109] Antoniotti C , Rossini D, Pietrantonio F, et al. FOLFOXIRI plus bevacizumab and atezolizumab as upfront treatment of unresectable metastatic colorectal cancer (mCRC): updated and overall survival results of the phase II randomized AtezoTRIBE study. J Clin Oncol. 2023;41(16_suppl):3500-3500. 10.1200/jco.2023.41.16_suppl.3500

[110] Sanz-Garcia E , Argiles G, Elez E, Tabernero J. BRAF mutant colorectal cancer: prognosis, treatment, and new perspectives. Ann Oncol: Off J Eur Soc Med Oncol. 2017;28(11):2648-2657. 10.1093/annonc/mdx401 29045527

[111] Tabernero J , Grothey A, Van Cutsem E, et al. Encorafenib plus cetuximab as a new standard of care for previously treated BRAF V600E-mutant metastatic colorectal cancer: updated survival results and subgroup analyses from the BEACON study. J Clin Oncol. 2021;39(4):273-284. 10.1200/JCO.20.02088 33503393 PMC8078423

[112] Sumimoto H , Imabayashi F, Iwata T, Kawakami Y. The BRAF-MAPK signaling pathway is essential for cancer-immune evasion in human melanoma cells. J Exp Med. 2006;203(7):1651-1656. 10.1084/jem.20051848 16801397 PMC2118331

[113] Cen S , Liu K, Zheng Y, et al. BRAF mutation as a potential therapeutic target for checkpoint inhibitors: a comprehensive analysis of immune microenvironment in BRAF mutated colon cancer. Front Cell Dev Biol. 2021;9:705060. 10.3389/fcell.2021.705060 34381786 PMC8350390

[114] Ghahremanifard P , Chanda A, Bonni S, Bose P. TGF-β mediated immune evasion in cancer—spotlight on cancer-associated fibroblasts. Cancers 2020;12(12):3650. 10.3390/cancers12123650 33291370 PMC7762018

[115] Zhang B , Halder SK, Zhang S, Datta PK. Targeting transforming growth factor-beta signaling in liver metastasis of colon cancer. Cancer Lett. 2009;277(1):114-120. 10.1016/j.canlet.2008.11.035 19147275 PMC2776056

[116] Guinney J , Dienstmann R, Wang X, et al. The consensus molecular subtypes of colorectal cancer. Nat Med. 2015;21(11):1350-1356. 10.1038/nm.3967 26457759 PMC4636487

[117] Kim B-G , Malek E, Choi SH, Ignatz-Hoover JJ, Driscoll JJ. Novel therapies emerging in oncology to target the TGF-β pathway. J Hematol Oncol. 2021;14(1):55. 10.1186/s13045-021-01053-x 33823905 PMC8022551

